# Quantitative Determination of Spring Water Quality Parameters via Electronic Tongue

**DOI:** 10.3390/s18010040

**Published:** 2017-12-25

**Authors:** Noèlia Carbó, Javier López Carrero, F. Javier Garcia-Castillo, Isabel Tormos, Estela Olivas, Elisa Folch, Miguel Alcañiz Fillol, Juan Soto, Ramón Martínez-Máñez, M. Carmen Martínez-Bisbal

**Affiliations:** 1 Instituto Interuniversitario de Investigación de Reconocimiento Molecular y Desarrollo Tecnológico (IDM), Unidad Mixta Universitat Politècnica de València-Universitat de València, Camí de Vera s/N, 46022 Valencia, Spain; noeliamorella@gmail.com (N.C.); javlopcar@gmail.com (J.L.C.); mialcan@upvnet.upv.es (M.A.F.); jsotoca@upv.es (J.S.); 2 Sociedad de Fomento Agrícola Castellonense (FACSA), C/Mayor 82-84, 12001 Castellón, Spain; jgarcia@facsa.com (F.J.G.-C); itormos@grupogimeno.com (I.T.); eolivas@facsa.com (E.O.); efolch@facsa.com (E.F.); 3 Departamento de Ingeniería Electrónica, Escuela Técnica Superior de Ingeniería del Diseño, Universitat Politècnica de Valencia, Camino de Vera s/N, 46022 Valencia, Spain; 4 Departamento de Química, Universitat Politècnica de Valencia, Camino de Vera s/N, 46022 Valencia, Spain; 5 CIBER de Bioingeniería, Biomateriales y Nanomedicina (CIBER-BNN), Instituto de Salud Carlos III, Spain; 6 Unidad Mixta de Investigación en Nanomedicina y Sensores, Universitat Politècnica de València, IIS La Fe, 46026 Valencia, Spain; 7 Unidad Mixta UPV-CIPF de Investigación en Mecanismos de Enfermedades y Nanomedicina, Universitat Politècnica de València, Centro de Investigación Príncipe Felipe, 46022 Valencia, Spain

**Keywords:** spring water, electronic voltammetric tongue, water quality control, partial least squares

## Abstract

The use of a voltammetric electronic tongue for the quantitative analysis of quality parameters in spring water is proposed here. The electronic voltammetric tongue consisted of a set of four noble electrodes (iridium, rhodium, platinum, and gold) housed inside a stainless steel cylinder. These noble metals have a high durability and are not demanding for maintenance, features required for the development of future automated equipment. A pulse voltammetry study was conducted in 83 spring water samples to determine concentrations of nitrate (range: 6.9–115 mg/L), sulfate (32–472 mg/L), fluoride (0.08–0.26 mg/L), chloride (17–190 mg/L), and sodium (11–94 mg/L) as well as pH (7.3–7.8). These parameters were also determined by routine analytical methods in spring water samples. A partial least squares (PLS) analysis was run to obtain a model to predict these parameter. Orthogonal signal correction (OSC) was applied in the preprocessing step. Calibration (67%) and validation (33%) sets were selected randomly. The electronic tongue showed good predictive power to determine the concentrations of nitrate, sulfate, chloride, and sodium as well as pH and displayed a lower R^2^ and slope in the validation set for fluoride. Nitrate and fluoride concentrations were estimated with errors lower than 15%, whereas chloride, sulfate, and sodium concentrations as well as pH were estimated with errors below 10%.

## 1. Introduction

The quality control of natural spring water is of interest and is a priority action of government authorities. Raw water plays an essential role for drinking purposes and for other sanitary uses and industrial processes [1]. With these aims in mind, national and international regulations have been developed to control the quality of drinking water [2]. In Spain, the reference values for the diverse water parameters (including both microbiological and physicochemical parameters) were established by a European law (Council Directive 98/83/EC of 3 November 1998 on the quality of water intended for human consumption), which was transposed into Spain’s law by the Royal Decree (RD) 140/2003. Drinking water is usually obtained from ground water, rivers, or reservoirs. Afterwards, the water is processed for human consumption and other purposes in treatment plants. Subsequently, the water is distributed alone or mixed with other natural waters depending on their composition to achieve a final balanced chemical profile in accordance with legal requirements. Water quality is assessed by routine analytical procedures, which are generally performed off-line and time-separated. It is usually assumed that water from natural sources such as springs and rivers has a constant composition. Nevertheless, sudden changes in the composition might occur due to accidentally contamination, floods, or geological events, which should ideally be detected before water enters to the treatment plant. To detect these potential sudden changes in water quality, the most commonly used systems are based on physicochemical measurements [3]. The use of systems able to report these changes in situ and online are desired to improve operations in water treatment plants. Traditional analytical methods can be used for water quality control, but they are expensive, are off-line, require trained personnel, and are time-consuming.

From another point of view, electronic tongues (e-tongues) have recently been introduced as a suitable tool applied to characterize the attributes of complex systems [4,5,6]. They are based in the use of unspecific sensors usually integrated in an array, and their response is commonly analyzed by suitable pattern recognition algorithms [4]. A vast number of e-tongues have been described based on different techniques, but the majority are based on electrochemical measurements [4]. Among the diverse electrochemical techniques that can be implemented in an electronic tongue, the most versatile and robust is perhaps voltammetry because it is usually less influenced by electrical disturbances and has a favorable signal-to-noise ratio [7]. Voltammetric e-tongues have been reported to be especially sensitive to changes in redox attributes. However, it has also been reported that the lack of clear redox processes does not necessarily hamper the use of voltammetric e-tongues since the presence of non-Faradaic effects due to chemical adsorption on the working electrode, or the influence that the presence of chemical species can have on the currents from the reduction or oxidation of water or on the equilibrium potential of the electrodes, might also be useful for multivariate data analysis [8].

E-tongues have found a number of applications in the classification of complex systems such as food and water samples [4,5]. In the field of drinking and natural waters, there are examples of the application of e-tongues based on various technologies and aimed at sensory detection, the classification of origin and brand, and the quantification of contaminants [9,10,11,12,13]. In the context of sensory detection, Taniguchi et al. developed a taste sensor containing lipid membranes to detect pollutants and unusual tastes in water to distinguish between normal tap water and tap water containing 50 ppm poly aluminum chloride, and these results correlated well with sensory tests [9]. Moreover, this system was also reported to distinguish normal river water from river water contaminated by 1 ppm free cyanide and 1 ppm of cyano complexes [9]. A commercial electronic tongue consisting of an array of seven liquid cross-selective potentiometric sensors was tested by Xiao et al. to classify three brands of bottled water and to monitor subtle quality changes in unsealed bottles that may be related to microorganism propagation [10]. In this case, the sensors used in the array contained an organic coating sensitive to certain species in the samples [10]. Different tap waters have also been successfully classified using a potentiometric e-tongue based on thick-film technology using an array containing different metallic, carbon, and RuO_2_ electrodes [11,12]. The discrimination between mineral waters from different geographical origins using sensory evaluation combining e-tongue measurements and chemical analysis of different ions has also been studied [14]. An e-tongue composed of an integrated array of six independent sensors based on diverse technologies was used to classify 13 different brands of mineral water and provided a 100% correct prediction [13].

There are more focused examples related to the quantitative determination of contaminants in water. With this aim, polymeric sensors based on impedance measurements have been tested to detect contaminant substances originated in the algae degradation process [15]. A sensor based on a voltammetric e-tongue containing gold and platinum as working electrodes has been reported to exhibit promising results for fast detection of qualitative changes in water quality and for the detection of fluoride, chlorine, and microbial contamination [16]. Finally, Winquist et al. used a voltammetric e-tongue containing gold, iridium, platinum, and rhodium as working electrodes for the detection of NaOCl, NaCl, and NaHSO_3_ in water samples [1].

The quantification of compounds in real complex samples as natural waters at low concentration is difficult. Nevertheless, there are several studies aiming at the quantification of diverse chemical species in drinking water. Contaminants as geosmin and methylisoborneol, which are subproducts from algae decomposition, have been quantified in distilled water and tap water in concentrations as low as 25 ng·L^−1^ using an e-tongue based on non-specific polymeric sensors and impedance measurements [15]. The quantitative determination of diverse organic acids at concentrations ranging from 0.01 to 0.1 M in water samples was recently achieved by Escobar et al. using an electronic voltammetric tongue [17]. We have recently reported the use of a voltammetric e-tongue able to quantitatively detect several water quality parameters such as ammonia, nitrates, phosphate, chemical oxygen demand and conductivity in real waste water samples [8,18].

Despite these reported examples, the quantitative determination of solutes in water using e-tongues is still challenging and advances in this research field could be of importance. As stated above, the quantitative determination of substances of interest in water is usually carried out by traditional laboratory analytical techniques, whose application to real-time monitoring and process controls is difficult [19]. Based on these concepts described above, we report herein a study on the ability of a simple voltammetric e-tongue, based on four noble metals, Ir, Rh, Pt, and Au, to determine nitrate, chloride, sodium, sulfate, and fluoride content as well as pH in spring water. The e-tongue was able to determine the concentrations of nitrate and fluoride with errors lower than 15%, whereas concentrations of chloride, sulfate, and sodium as well as pH were estimated with errors below 10%.

## 2. Materials and Methods

### 2.1. Spring Water Sample Collection and Analytical Measurements

Water samples from nine Spanish natural springs were studied. Each spring water sample underwent complete chemical and microbiological analysis (IPROMA Castellón Exp.: 103/LE268). Analytical and microbial analysis determinations were performed using routine procedures. According to the established norm and the expected ranges, nitrate, sulfate, fluoride and chloride concentrations were determined by ionic chromatography, whereas sodium content was determined by inductively coupled plasma atomic emission spectroscopy. pH was determined with a standard pH-meter. The number of colony-forming units (cfu) of *Escherichia coli*, *Enterococcus*, and *Clostridium perfringens* were determined by filtration counting. All the samples contained 0 cfu of the microorganisms studied. Table 1 shows the analytical parameters and the pH of each spring.

According to the chemical and microbiological analysis of the samples and the reference values in the Spanish Royal Decree (RD) 140/2003, the waters from Springs 5–9 were classified as potable, and the waters from Springs 1–4 were classified as non-potable (see spring numbers in Table 1).

From these nine springs, a total of 83 samples were collected in a period of two months. An invariable composition along this period of time was assumed for each spring. The number of samples studied for each spring is shown in Table 1. Once the samples were collected, they were stored at 5.0 °C until they were analyzed using the e-tongue.

### 2.2. Voltammetric e-Tongue Measurements

**Figure 1:**
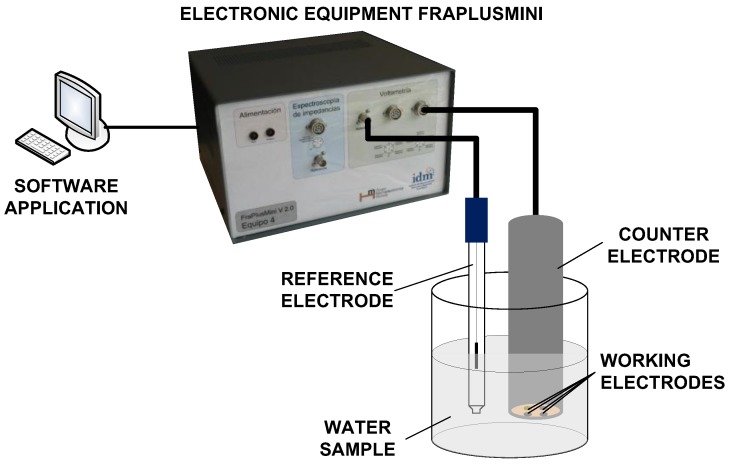
Block diagram of the measurement system.

The e-tongue system used in this study was based on pulse voltammetry, and was developed by the Instituto Interuniversitario de Investigación de Reconocimiento Molecular y Desarrollo Tecnológico (IDM), at the Universitat Politècnica de València (UPV, Valencia, Spain). The e-tongue was composed of an electronic equipment, a software application that runs on a personal computer (PC), and a set of metallic electrodes. The electronic equipment applies voltage signals to electrodes. The temporal evolution of the current signal was collected for each working electrode and sent to the PC to be stored for further processing. A set of pulses was put together to form a pulse train in order to extract as much information as possible from the solution [20]. The details of this electronic equipment have been published elsewhere [21]. Following the methodology proposed by Winquist et al. [22], the e-tongue device used in this work consisted in an array of four metallic working electrodes, Ir, Rh, Pt, and Au, with a purity of 99.9% and a 2 mm diameter. The noble metals used in the working electrodes show high durability, owing to their low reactivity. Nevertheless, despite their low reactivity, these electrodes can still provide information and are less maintenance demanding than the non-noble metals, as desirable to develop future automated equipment. In fact, their suitability for the quantitative analysis of quality parameters in wastewater has already been shown [18]. The electrodes were housed inside a homemade stainless steel cylinder, which was used at the same time as both the body of the e-tongue system and the counter electrode. A more detailed description of the electrodes used can be found in [8,23]. Electrodes were conditioned before the measurements were taken by mechanical polishing and immersion in an acidic solution. The electrodes were then rinsed with distilled water before the measurements were performed. A saturated calomel electrode was used as the reference electrode. A scheme of the e-tongue is shown in Figure 1.

**Figure 2:**
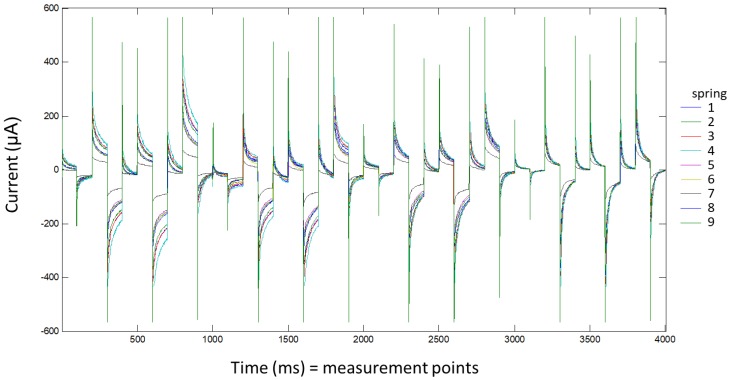
Responses from single examples of each spring included in the analysis. The data of all the electrodes are shown in the same figure. The abscissa axis shows the measured points and is equivalent to the time in ms as a pulse is described with 100 points and a duration is 100 ms.

For each sample, three aliquots were measured with a voltammetric e-tongue. For each measurement, 50 mL of water were introduced into a cell and thermostatted at 24.0 ± 0.1 °C (PolyScience). Voltammetric measurements were taken without adding any background electrolyte. The applied pulse sequence was the same for all four working electrodes and was composed of 10 pulses: 200, 0, 600, −500, 0, 400, −750, 0, 750, and 150 mV. Each pulse was applied for 100 ms. Current values collected per pulse and electrode were described by 100 measurements (1 data/ms). These pulses were designed according to the cyclic voltammetry information obtained in a previously published work [8]. In all samples, 4000 points (10 pulses × 100 points for pulse × 4 electrodes) were recorded. The voltagrams of one example of each spring are shown in Figure 2.

### 2.3. Data Management and Statistical Analysis

The average of the data from the three aliquots was calculated for each of the 83 water samples. As mentioned above, 4000 points were recorded in each voltammetry experiment. A multivariate analysis was performed by using partial least squares method (PLS) [24] and using the software *PLS_Toolbox Solo 8.0* (Eigenvector Research, Inc., Manson, WA, USA) for chemometrics analysis.

Before the analysis, data were preprocessed. Orthogonal signal correction (OSC) and autoscaling was applied to X-block (voltammetry data). OSC was introduced to remove one or more directions in X that are orthogonal to Y, and these directions account for the largest variation in X. Thus, the removal of information that is important for prediction was avoided [25,26]. The main idea in OSC is to reduce the variation in X not correlated to Y [26]. OSC was performed as a preprocessing step prior to latent variable regression modeling to improve the calibration model [25,27]. OSC has been already applied in the preprocessing of near-infrared spectroscopy [25,26] and on electrochemical data [27,28,29]. In particular, Rouhollahi et al. used OSC in linear sweep voltammetry data to improve predictions of the determination of ascorbic acid (in concentrations ranging from 3 to 350 ppm) and dopamine (ranging from 5 to 350 ppm) with a glassy carbon electrode [27], resulting in lower RMSEP values for the content prediction of both acids, compared to the performance of the model calculated with PLS without OSC. In another study, OSC preprocessing improved the resolution of quaternary mixtures of phenols analyzed by differential pulse adsorptive stripping voltammetry [29]. In this case, OSC correction helped to remove the differences between samples due to changes in the experimental conditions along time in which the measurements were performed. Another example was the improvement of the simultaneous determination of cinnamic acid and 2,4-dihydroxy benzoic acid after OCS processing of cyclic voltammograms taken in a glassy carbon disc electrode [28]. In this work, the evaluation of the prediction errors for the validation set revealed that the OSC processed data gave substantially lower RMSD values than those of the original data, whereas at the same time OSC-filtered data provided much simpler calibration models with fewer components than the ones based on the original data [28].

Following with the preprocessing, after performing OSC, the data were autoscaled in an operation in which each variable (column) of the data was normalized to the standard deviation of that column. In addition, the mean of the column was subtracted. The result was that each variable had a mean of zero and a standard deviation of one.

The data were split into calibration and validation sets, and the samples of each spring, including 56 samples (≈67%) in the calibration set and 27 samples (≈33%) in the validation set, were randomly selected. The same calibration and validation sets were used to obtain the predictions for all the parameters. In order to evaluate the adequacy of the experimental data and to select the quantity of latent variables, cross-validation (CV) was performed before building the model. CV random subsets was the method selected for the CV, whereby the calibration set was divided into 10 subsets and 20 iterations were performed. The minimum value for the CV error was used to select the number of latent variables used to calculate the model for each parameter. Then, each model was applied to the validation set to predict the values of the parameters: i.e., the concentrations of nitrate, chloride, sodium, sulfate, and fluoride as well as the pH level of the solution. The models were evaluated to determine the overfit risk by running permutation tests (the pairwise Wilcoxon, the signed rank test, and the Rand t-test) 100 times. Finally, models were evaluated by comparing real versus predicted concentrations using the correlation coefficient (R^2^), the root mean square error of prediction (RMSEP), and the slope (p1) and the intercept (p2) of the validation set (from y = p1·x + p2 based on a simple linear model).

The range of content for chloride and sodium was between 17 and 190 ppm and between 11 and 94 ppm, respectively. However, the content in the samples was not distributed equally along the data range. Spring 4 had extreme values for chloride (190 mg/L) and sodium (94 mg/L), as can be observed in Table 1. These values were far from the range that included the rest of the values (17–66 mg/L for chloride and 11–41 mg/L for sodium). To test that the predictions were robust in the most habitual range and that the linearity was not governed by the higher levels of chloride and sodium in Spring 4, additional models for prediction were calculated with the same method excluding Spring 4 from the calibration and validation sets. The new data sets are named Chloride II and Sodium II in Table 2.

Taking into account the overall quality of the samples, a multivariate analysis was performed using the PLS discriminant analysis method (PLS-DA) and using the software *PLS_Toolbox Solo 8.0* (Eigenvector Research, Inc., Manson, WA, USA) to study the ability of the system to discriminate between the samples according to its quality. The samples were classified as potable (Springs 5–9) or non-potable (Springs 1–4), taking into account the Spanish law and the chemical and microbiological analysis. The voltammetric data underwent the same procedures as those in the previous PLS analysis for preprocessing, calibration and validation set splitting, and CV. The same calibration and validation sets were considered. A model was calculated using the data from the calibration set. This model was then applied to the validation set to predict the samples as being potable or non-potable. The model was evaluated to determine the overfit risk by running the permutation test 100 times. Finally, the model evaluation was made by determining the sensibility and specificity according to the samples correctly classified and the misclassifications.

## 3. Results and Discussion

A PLS analysis was used to obtain a correlation between voltammetric measurements using the e-tongue and the concentrations of nitrate, chloride, sodium, sulfate, and fluoride, according to analytical tests, as well as pH. A model was calculated for each quality parameter according to the data in the calibration set. Afterwards, the model was applied to the validation set to evaluate the model performance. Additional models were calculated to predict chloride and sodium concentrations excluding Spring 4 (sets Chloride II and Sodium II), which presented atypically elevated values (vide infra). The values of R^2^, RMSEP, and p1 and p2 for the validation set are shown in Table 2 for all parameters and for Chloride II and Sodium II, together with the range of measurements. Just to provide an idea of the error in the predictions, the RMSEP in relation to the maximum value within the range of each parameter was calculated as a percentage and is also shown in Table 2. The evaluation determining the overfit risk performed by running permutation tests (the pairwise Wilcoxon, the signed rank test, and the Rand *t*-test) showed that, in all cases, the probability of model insignificance versus permuted samples was lower than 0.05, indicating that the models were significant at the 95% confidence level.

**Figure 3:**
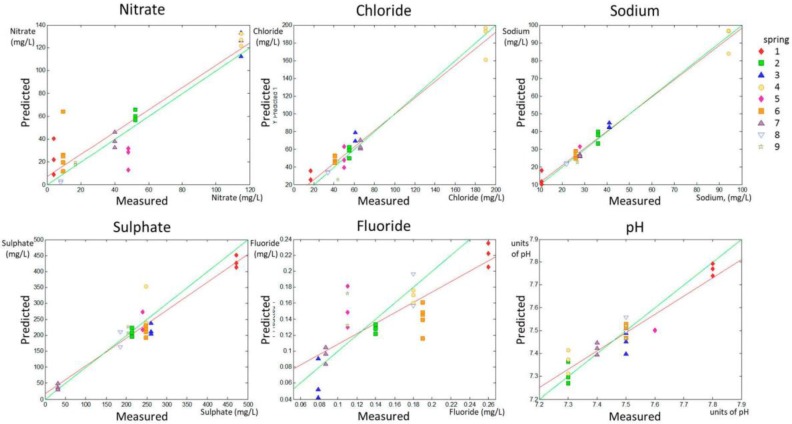
PLS prediction for the validation set for nitrate, chloride, sodium, sulfate, and fluoride content as well as pH. The green line is the line 1:1, whereas the red line is the fitted line y = p1·x + p2 for the validation set.

The PLS prediction set for the whole range of the different quality parameters are shown in Figure 3 (predicted versus measured parameter). Figure 3 allows for a visual inspection of the accuracy and precision of the model applied to the validation set.

**Figure 4:**
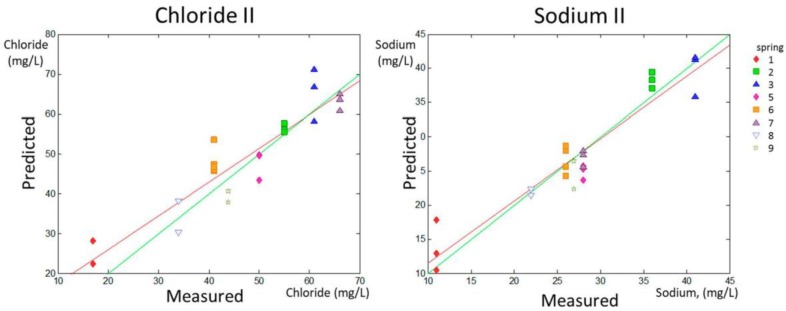
PLS prediction for the validation set for Chloride II and Sodium II. In both cases, for this calculation, the spring with the higher values was not considered for training and validation. The green line is the line 1:1, whereas the red line is the fitted line y = p1·x + p2 for the validation set.

The PLS prediction sets for Chloride II and Sodium II without considering Spring 4 are shown in Figure 4 (predicted versus measured parameter).

A numerical evaluation of the accuracy and precision of predictions can be obtained by linearly fitting the experimental points calculated in the predicted group according to the model in the calibration set (red solid line in the predicted versus measured parameter graphic for each parameter shown in Figure 1 and Figure 2). The slope (p1) and the intercept with the *y*-axis (p2) of the linear fitting of the predicted vs. real data in the validation set were related to accuracy in prediction. The model is much better as p1 approaches 1 (green lines in Figure 1 and Figure 2 represent p1 = 1). RMSEP deals with model precision and the model is much better for prediction as RMSEP approaches 0 (that would occur if all predicted points were in the red line in Figure 1 and Figure 2).

The data in Table 2 indicate that the predictions for nitrate, sulfate, chloride, and sodium concentrations, as well as Chloride II and Sodium II, yielded p1 values higher than 0.8, whereas the p1 for pH determination was 0.799, indicating fairly high accuracy in the prediction of these parameters. Fluoride prediction was the only parameter that showed a p1 much lower than 0.8 (0.653). Regarding the precision of the results, this experimental setting allowed for the prediction of the concentrations of nitrates and fluoride with an approximate error of 15% (14.78% and 11.54% respectively), and for the prediction of the concentrations of chloride, sulfate, and sodium and of the pH value with an error lower than 10%, according to the RMSEP ratio to the higher value in the range for each parameter, shown in Table 2.

**Figure 5:**
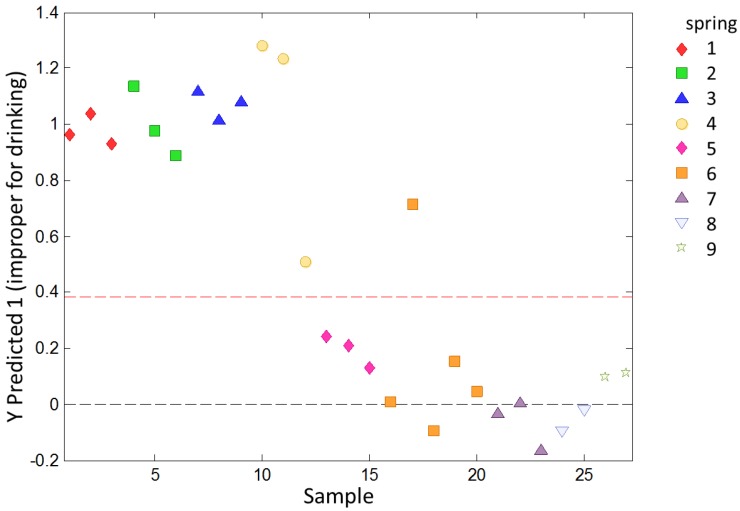
PLS-DA prediction for the validation set for classifying water samples as potable or non-potable. Springs 1–4 contain non-potable water, and Springs 5–9 contain potable water. The dashed red line indicates the limit beyond discrimination between potable (down) and non-potable (up) samples cannot occur.

To study the ability of the e-tongue to classify water samples with different levels of overall quality, a PLS-DA analysis was used. The samples were previously classified as potable or non-potable according to chemical and microbiological analysis. The model calculated according to the data in the calibration set was applied to the validation set. The samples in the validation set were classified as shown in Figure 5.

Except for one sample from Spring 6, all samples were correctly classified. The sensitivity on the prediction for this model was 1.000 and the specificity was 0.933. The evaluation to determine the overfit risk by running permutation tests showed that the probability of model insignificance versus permuted samples was lower than 0.05, indicating that the model was significant at the 95% confidence level.

As far as we know, this is the first study on the quantitative determination of several chemical species, of interest for water quality control, using a voltammetric e-tongue. In the spring waters studied, the range of variation in the quality parameters were quite small, especially for pH and fluoride concentration (ranging from 0.05 to 0.30 mg/L and from 7.3 to 7.8, respectively). Nevertheless, the models worked satisfactorily for the prediction of the concentration of most of the specimens from the studied springs. Moreover, the same voltammetric data provided a model for classifying the waters according to the overall quality as potable or non-potable.

## 4. Conclusions

The use of a simple electronic tongue is described herein for spring water quality parameter analysis and for classification as potable or non-potable water. The parameters considered in this study (pH as well as nitrate, sulfate, fluoride, chloride, and sodium concentrations) provide an idea of the quality of the spring water, as their concentrations and values are important if decisions on the treatment of the waters or on its distribution for human consumption need to be made. The e-tongue employed in this study used four noble electrodes (iridium, rhodium, platinum, and gold) housed inside a stainless steel cylinder. These noble metals have high durability and low reactivity, require less maintenance than non-noble metals, and have already shown their suitability for the quantification of quality parameters in wastewater. A pulse voltammetry study was conducted in 83 spring water samples to determine the concentration of nitrate, sulfate, fluoride, chloride, and sodium as well as the pH. These parameters were also measured by routine analytical methods. Calibration (67%) and validation (33%) sets were selected randomly among the spring water samples. The electronic tongue was able to predict the concentration of nitrate, sulfate, chloride, and sodium as well as the pH with errors lower than 10%. A higher number of samples of diverse composition and the study of the redox processes related to the quality parameters when the pulse train is implemented might improve predictions. Nevertheless, these results, in terms of accuracy and precision, suggested that this sensing strategy could be of use for the fast quality control of spring waters, which is usually the source of water for drinking, sanitary, and relevant industrial purposes. Moreover, the same system provided high sensitivity and specificity in the discrimination between waters potable or non-potable. Usually, a regular composition of the spring waters is assumed, and quality controls are performed via conventional analytical methods in a laboratory. In contrast, methods based in e-tongues, such as the one we report here, might not be as precise as standard protocols, but they are simple, robust, and low-cost and can work in situ and online for the instantaneous detection of changes in spring water composition.

## Figures and Tables

**Table 1:** Number of samples analyzed, nitrate, chloride, sodium, sulfate, and fluoride concentrations (in mg/L), and pH values for each natural water spring studied.

Spring	Samples	Nitrate ^1^	Chloride ^1^	Sodium ^1^	Sulfate ^1^	Fluoride ^1^	pH
1	12	3.9	17	11	472	0.26	7.8
2	9	52	55	36	214	0.14	7.3
3	11	115	61	41	261	0.08	7.5
4	5	115	190	94	248	0.18	7.3
5	9	48	50	28	240	0.11	7.6
6	9	9.5	41	26	248	0.19	7.5
7	9	40	66	28	32	0.09	7.4
8	10	8.1	34	22	185	0.18	7.5
9	9	17	44	27	205	0.11	7.5

^1^ The content of ions is shown in mg/L.

**Table 2:** The range of data for different quality parameters. R^2^, RMSEP, and p1 and p2 data for the different parameters. CV random subsets, 10 subsets, 20 iterations.

	Range	R^2^	RMSEP	p1	p2	RMSEP × 100/max
pH	7.3–7.8	0.856	0.1	0.799	1.50	1.28
Nitrate	3.9–115	0.852	17	0.973	7.15	14.78
Sulfate	32–472	0.898	36	0.876	16.00	7.63
Fluoride	0.08–0.26	0.630	0.03	0.653	0.04	11.54
Chloride	17–190	0.955	10	0.920	7.36	5.26
Chloride II	17–66	0.869	6	0.845	9.15	9.09
Sodium	11–94	0.980	3	0.967	1.46	3.19
Sodium II	11–41	0.895	3	0.911	2.25	7.32
